# Applying affect coding and dynamical systems mathematical modeling to understanding the role of emotional expression on the therapeutic relationship across an entire course of substance abuse treatment

**DOI:** 10.3389/fnhum.2025.1544437

**Published:** 2025-04-23

**Authors:** Gina Cipriano, Paul R. Peluso, Emma Bright, Blanka Hutarkova

**Affiliations:** ^1^Department of Counselor Education, Florida Atlantic University, Boca Raton, FL, United States; ^2^Department of Human Development and Family Science, Florida State University, Boca Raton, FL, United States

**Keywords:** alliance, dynamical systems (DS), emotional expression, therapeutic relationship, substance abuse treatment, affect coding

## Abstract

**Introduction:**

Substance abuse remains a critical public health issue, with 48.7 million adults in the United States meeting the criteria for a substance use disorder (Substance Abuse Mental Health Services Administration [SAMHSA], 2023). Traditional substance abuse treatment is often considered distinct from other psychotherapeutic approaches. Practitioners have historically focused on compliance and behavior arrest rather than exploring underlying issues. Despite these efforts, relapse rates for substance abuse remain high, prompting the development of alternative treatments incorporating psychotherapeutic methods such as Motivational Interviewing and various mindfulness-based harm reduction. This paper reviews Alan Marlatt's mindfulness-based approach to substance abuse treatment, which emphasizes the therapeutic relationship's role in reducing resistance and enhancing client autonomy. The findings aim to improve therapeutic outcomes by providing a deeper understanding of these emotional interactions, ultimately contributing to more effective substance abuse interventions.

**Method:**

This study utilized the APA-produced DVD series *Psychotherapy Over Time*, featuring Dr. Alan Marlatt and his client, Kevin, over six therapy sessions. The sessions were coded using the Specific Affect Coding System (SPAFF) to code emotional expressions and a dynamical systems (DS) mathematical model, with parameters derived from the coded data to create unique models for each session.

**Results:**

Statistical analysis was used to compare SPAFF codes and model parameters between Alan Marlatt and his client. The therapist showed significant changes in several affect codes (e.g., Low Domineering and Sadness) as did the client (e.g., Disgust, Contempt) over six sessions. Despite these differences, the overall model parameters remained stable across the six sessions.

**Discussion:**

This study utilized SPAFF coding and DS modeling to analyze emotional expressions between Dr. Alan Marlatt and his client, over six psychotherapy sessions focused on relapse prevention. The results revealed consistent emotional expressions from Marlatt, while Kevin exhibited significant fluctuations, reflecting his struggles with addictions and relapse. Despite these variations, the overall model parameters remained stable, indicating a consistent therapeutic relationship. These findings highlight the complex emotional dynamics in substance abuse treatment and underscore the importance of a stable therapeutic presence.

**Clinical significance/impact statement:**

The findings from this study highlight the importance of understanding emotional dynamics in the therapeutic relationship during substance abuse treatment. The significant variations in Kevin's emotional expressions across sessions, contrasted with the stability of Marlatt's responses suggests that consistent therapeutic presences can provide a stable foundation for clients experiencing fluctuating emotional states. By employing affect coding and dynamical systems modeling, this research underscores the potential for these methods to enhance therapeutic outcomes through a deeper understanding of client-therapist interactions. These insights can inform the development of more effective, emotionally responsive treatment protocols, ultimately improving recovery rates and reducing relapse in substance abuse therapy.

## Introduction

The rates of substance abuse remain alarmingly high. According to the Substance Abuse and Mental Health Services Administration (SAMHSA), 48.7 million adults in the United States meet the criteria for a substance use disorder (Substance Abuse and Mental Health Services Administration, 2023). As a result, the treatment of substance abuse continues to be one of the most prevalent clinical issues that clinicians need to address. Unlike other disorders, where the applications of psychotherapy are generally not differentiated, substance abuse treatment is often considered to be distinct from other psychotherapeutic treatments (Knuuttila et al., 2012; Wampold and Imel, 2015). Throughout most of the twentieth century, the history of substance abuse treatment was dominated by either a medical interventionist approach, or the 12-step recovery group treatment model. These often seemed to be at odds with the psychotherapy approaches of the day (Paquette et al., 2022). While much of focus of psychotherapy was on exploring the underlying issues that produced symptoms or perpetuated a disorder, the focus of substance abuse treatment was on compliance with a program of treatment to arrest the behavior (namely, using drugs or alcohol; Howard et al., 2012; Norcross and Lambert, 2019; Weinman, 2011). Unfortunately, compared to the efficacy demonstrated by other psychotherapeutic approaches, substance abuse treatment did not seem to keep pace. According to the National Institute for Drug Abuse, on average 40 to 60 percent of individuals relapse within the first year, and—depending on the substance being abused—the rate can go as high as 85% (Brandon et al., 2007; National Institute on Drug Abuse, 2023). This led to the rise of alternative approaches to substance abuse treatment in the last three decades that incorporated more traditional psychotherapeutic approaches (i.e., cognitive-behavioral therapy), such as Miller and Rollnick's (2012) Motivational Interviewing, and the mindfulness based relapse prevention and relapse prevention model pioneered by Alan Marlatt (Marlatt, 2006; Witkiewitz et al., 2005).

These modern approaches to substance abuse treatment have demonstrated greater efficacy in treating clients with substance abuse in a number of studies on par with findings in psychotherapy outcome studies (Chiesa and Serretti, 2013; DiClemente et al., 2017; Wampold and Imel, 2015). One of the hallmarks of these approaches has been the reliance on the quality of the therapeutic relationship between the therapist and client, which was not generally emphasized by traditional substance abuse treatment, previously (Tatarsky and Marlatt, 2010; Miller and Rollnick, 2012). For example, Motivational Interviewing is centered on the idea of reducing resistance by emphasizing clients' autonomy in formulating treatment goals, while mindfulness-based relapse prevention utilizes the therapeutic relationship to encourage compassionate-based inquiry and decrease self-criticism similar to other psychotherapeutic approaches (Garland and Howard, 2018; Marlatt et al., 2012; Paquette et al., 2022). This paper will review Marlatt's mindfulness-based approach to substance abuse treatment and use a method for evaluating the emotional dynamics between therapist and client that has previously been used to evaluate the quality of therapy relationships (Baker et al., 2022; Diaz et al., 2023; Luedke et al., 2017; Peluso et al., 2012).

### Mindfulness-based and harm reduction substance abuse treatment

In 1985, Marlatt created a cognitive behavioral model of relapse prevention to assist those with substance use issues (Witkiewitz and Marlatt, 2004). In this approach, relapses are used as opportunities to encourage better relapse management. Witkiewitz and Marlatt's (2004) dynamic model of relapse includes prior predispositions and present moment cognitive, affective, and coping responses as factors that can impact potential relapse. Through the lens of relapse prevention, Witkiewitz and Marlatt (2004) theorized that “abstinence violation effects” (AVE) may occur, and if the client is not prepared to manage it, a lapse is likely to happen. In addition, clients' negative affective states associated with the lapse and unhelpful cognitions may contribute to lapses becoming ongoing and progressive versus a one-time event. Thus, within the relapse prevention model, compassionately addressing the guilt and shame following lapses becomes a method for addressing the AVEs and potential future ones (Witkiewitz and Marlatt, 2004). Therapists provide clients with psychoeducation on maladaptive thought processes such as cognitive distortions. They then utilize cognitive and behavioral interventions such as cognitive restructuring and modeling to increase client's self-efficacy surrounding the management of future lapses.

Marlatt refined his approach and introduced Mindfulness Based Relapse Prevention (MBRP) to provide people who use substances with mindfulness skills to enhance their ability to observe their present-moment experience while enhancing compassion (Marlatt et al., 2012). According to Garland and Howard (2018):

mindfulness of one's automatized behavioral and emotional reactions may allow for greater self-regulation of habitual addictive behavior. Thus, mindfulness practice may evoke the state of mindfulness that accrues with each meditation practice session into a durable propensity to exhibit the trait of mindfulness in everyday life, thereby suffering as a buffer against addictive behavior (pp. 2–3).

MBRP allows clients to address thoughts and feelings that relate to relapse risks. It also encourages practitioners to avoid resisting clients' negative affect so that they can increase clients' threshold to cope with a range of emotions and thoughts, rather than trying to alter them (Marlatt et al., 2012).

A contrasting philosophy to traditional substance abuse treatment, harm reduction is guided by the principle understanding and addressing the person and their substance abuse in the context of their social environment. The primary aim of harm reduction is to reduce maladaptive behaviors rather than focusing solely on the eradication of symptoms and immediate cessation of all substance use (Logan and Marlatt, 2010). Unlike traditional substance abuse treatment, where treatment is considered successful through abstinence, the goal of this approach is either abstinence or moderation depending on identified goals collaborated between client and therapist. One of the pitfalls of traditional substance abuse treatment is that therapists may develop a “righting reflex” where they tell a client how to change, which can inadvertently lead to values imposition and/or contribute to the client becoming defensive and compromise the therapeutic relationship (Norcross and Wampold, 2019; Substance Abuse Mental Health Services Administration, 2019). Harm reduction enables the therapist to work with the client rather than against through eliciting the client's individual goals, motivations, and strengths (Tatarsky and Marlatt, 2010). As a result, the therapist models acceptance and non-judgment within the therapeutic relationship to facilitate clients' own curiosity about their maladaptive behaviors. Additionally, the stigmatization of substance abuse is challenged, and the client is seen from a strengths-based point of view, and that these strengths can be marshaled to help make sensible changes (Logan and Marlatt, 2010; Tatarsky and Marlatt, 2010). The principles of harm reduction can be utilized in conjunction with other treatment approaches including Witkiewitz and Marlatt's (2004) cognitive behavioral therapy-based relapse prevention and mindfulness-based relapse prevention (Marlatt et al., 2012).

### Therapeutic relationship, therapeutic alliance and substance abuse treatment

A long-standing tenet of the practice of psychotherapy is that the therapeutic relationship is an important, active element in therapeutic success (Norcross and Lambert, 2019; Wampold and Imel, 2015). Most recently, Flückiger et al. (2019), reported several meta analyses of one aspect of the therapeutic relationship, the therapeutic alliance, and its relationship to clinical outcomes. They found a medium effect size (*r* = 0.28) for psychotherapy in general, which replicated previous findings. In particular, they commented that the alliance is determined by “creating a *warm emotional bond* or collaborative attachment with the patient” (p. 61, *italics added*). Within the field of substance use, the therapeutic relationship is associated with increased treatment engagement (Campbell et al., 2015; Goldberg et al., 2020), decreases in psychological distress (Urbanoski et al., 2012), and increased treatment satisfaction (Knuuttila et al., 2012). Clients with substance use attributed a strong therapeutic alliance to therapists' flexibility to address a myriad of emotional topics rather than solely focusing on substance use, ability to negotiate treatment goals, and assistance in increasing client motivation toward their defined goals (Allen and Olson, 2015).

At the same time, the empirical base for the impact of the therapeutic alliance on abstinence is mixed (Knuuttila et al., 2012). Flückiger et al. (2019) in their same set of meta analyses found that when they isolated client-therapist relationships for the treatment of substance use disorder, there was a smaller effect size on outcomes compared to psychotherapy in general (*r* = 0.14), though they noted that there may be several methodological issues that account for the difference (i.e., the use of dropout or relapse as a measure of outcome vs. symptom reduction). Urbanoski et al. (2012) found that the therapeutic alliance was associated with decreases in psychological distress, but it did not account for changes in motivation, self-efficacy, coping skills, and commitment to 12-step meeting attendance (AA/NA). Instead, they discovered that client motivation at the start of treatment had the most significant effect on outcome. They speculated that this could be due to an “alliance-outcome” effect meaning that the outcome of therapy (i.e., client success/motivation) impacts the alliance and vice versa.

Knuuttila et al. (2012) found that client's rating of the working alliance at the first and third session and therapist's rating of the third session was associated with client's increased treatment satisfaction, but it did not predict abstinence. Similar to Urbanoski et al. (2012), they postulated that the alliance and treatment outcomes may have a bi-directional effect between treatment outcomes and perception of the alliance. Lastly, Maisto et al. (2015) found that there was no correlation between baseline self-efficacy measures and ratings of the therapeutic alliance, but at 3- and 9-month follow-up, there was a significant correlation between measures of the alliance, self-efficacy, and both percent of days drinking and days of drinking. Hence, regardless of any possible intervening effects, many modern-day substance abuse treatment clinicians and researchers now embrace the use of harm reduction techniques along the lines of Marlatt's approach, so therapeutic success is contingent on clients' abilities to remain engaged in treatment with the goal of reducing overall problematic use (Cook et al., 1995; Campbell et al., 2015; Goldberg et al., 2020; Marlatt et al., 2012).

### Emotional expression in the therapeutic relationship

Over the past two decades, the American Psychological Association (APA) has supported a task force to investigate different aspects of the therapeutic relationship. As part of the most recent iteration of this project, Peluso and Freund (2019) conducted several meta analyses examining the impact of emotional expression between therapists and clients in therapy on the therapeutic relationship as well as on the outcomes in therapy. They found significant medium effect sizes for emotional expression and the therapeutic process for clients (*d* = 0.63) and for therapists (*d* = 0.54). When they considered the relationship between emotional expression and outcomes, they found a medium effect size for therapist emotional expression and therapeutic outcome (*d* = 0.56), and a medium to large effect size for client emotional expression and therapeutic outcome (*d* = 0.85). At present, there is a lack of research on how therapists actively utilize these emotional expressions to create effective therapeutic relationships, and exactly how this related to positive therapeutic changes, prompting researchers to call for more research into this area (Baker et al., 2022; Diaz et al., 2023; Caspar, 2017; Hill et al., 2017; Peluso and Freund, 2019; Wampold et al., 2017). Given the positive effect that emotional expression seems to have on therapeutic process and outcome, as well the unique characteristics of the therapeutic relationship in mindfulness-based substance abuse treatment, an investigation into this dynamic would be warranted and beneficial.

### Use of dynamical systems to assess relationship dynamics in psychotherapy

One of the concluding recommendations from the APA task force was that future research into the therapeutic relationship should focus on aspects of the relationship at the second-by-second basis level, and not just at the end of a session or the conclusion of therapy (Norcross and Wampold, 2019). Furthermore, the task-force suggested that researchers consider several other factors, including using research designs that investigate more complex interactions, including observational methods (as opposed to relying on subjective measures), and focusing on the therapists' contributions to the therapeutic relationship. Dynamical systems (DS) is a research paradigm that measures complex phenomena, like relationships, that change over a period of time (Baker et al., 2022; Diaz et al., 2023; Liebovitch et al., 2011). Baker et al. outlined two areas of research with the therapeutic relationship where DS held particular promise of fulfilling Norcross and Wampold's (2019) suggestion: the range of elements of the therapeutic relationship that could be studied (e.g., emotional exchanges, word choices, coordination of movement), and the time-span under investigation (ranging from second-to-second, to session-to-session, and beyond). In fact, Peluso and Freund (2019) recommended that such modeling could “provide a rich graphical description of the dynamics of the relationship to therapists and researchers alike” (p. 449). Tschacher and Haken (2020) further detailed how DS approaches can successfully model deterministic features (i.e., attractors), as well as the stochastic (changing) elements within the dyad, providing a rich picture of therapeutic processes.

Most recently, researchers have begun to apply DS modeling to the therapeutic relationship. Following work by Peluso et al. (2012), Baker et al. (2022) used DS modeling and an affect coding system with actual therapy sessions conducted by three expert therapists from separate theoretical orientations (cognitive-behavioral therapy, emotion-focused therapy, and psychodynamic therapy). These experts (Judith Beck, Leslie Greenberg, and Nancy McWilliams) saw the same two clients for a total of six sessions which were then coded and modeled using DS equations. Specifically, they found: (1) expert therapists construct their therapeutic relationship dynamics similarly for the same client rather than based on the particular school of therapy and (2) DS mathematical modeling could be used to accurately capture and explore the emotional exchanges of the therapeutic relationship. These findings provided additional evidence to support the concept of the therapeutic relationship as a common factor rather than a specific ingredient based in a theoretical approach, and for the tailoring or relationships based on the client (Baker et al., 2022; Norcross and Lambert, 2019; Wampold and Imel, 2015).

Next, Diaz et al. (2023) followed up on Baker et al. (2022) and applied the same approach to a complete course of six sessions using the APA video series, *Psychotherapy Over Time* featuring Dr. Jon Carlson. Their findings replicated and extended Baker et al.'s work using DS modeling and affect coding. They found that an expert therapist (Carlson) showed stability in affect codes over six sessions while the client's affect codes appeared to be more flexible over time. At the same time, phase space portraits depicted the evolution of the affective dynamics between the master therapist and his client as the relationship matured, though the model parameters remained stable across the six sessions. In their conclusions, both Baker et al. and Diaz et al. recommended following up their findings by applying DS mathematical modeling to a full course of therapy, including specific diagnoses or conditions (like substance abuse), and consider how expert therapists develop the therapeutic relationship over time.

### Current study

Given the importance of substance use treatment, and the use of mindfulness-based harm-reduction approaches, as well as the ongoing questions about the impact of the therapeutic relationship in the treatment of individuals with substance use disorders, using novel approaches may yield important findings concerning the therapeutic relationship between clients and therapists in the treatment of substance abuse. In order to accomplish this, the current study aims to build upon the work of Peluso et al. (2012), Baker et al. (2022), and Diaz et al. (2023) by applying the same affect coding system (SPAFF) and dynamical systems modeling to a course of six psychotherapy sessions featuring G. Alan Marlatt utilizing relapse prevention to assist a client trying to address their substance abuse issues. We will examine the following research questions:

*Research Question 1*- How does emotional expression for both the therapist and client change across the entire course of therapy for substance abuse (as measured by SPAFF observational codes). Given the lack of consensus on the subject, this hypothesis is non-directional.
*Research Question 2*- How do the emotional dynamics of the relationship for both the therapist and client change across the entire course of substance abuse therapy (as measured by the DS mathematical model parameters). Again, given the lack of consensus on the subject, this hypothesis is non-directional.
*Research Question 3*- How does the overall therapeutic relationship change across the entire course of therapy? As this will be depicted by phase-portraits that will graphically represent the mathematical models across the six sessions, this will be a qualitative analysis of each of the portraits to examine how each session is similar or differs from one another [similar to Baker et al.'s (2022) and Diaz et al.'s (2023) analyses].

## Method

### Participants

The APA-produced DVD series *Psychotherapy Over Time* featured Dr. Alan Marlatt, and his client, Kevin, and their course of psychotherapy over six sessions. This was used for coding and mathematical modeling. Permission to use the APA published *Psychotherapy Over Time* series was obtained from The American Psychological Association for research purposes (G. VandenBos, personal communication, June, 13, 2014).

#### Therapist

Dr. G. Alan Marlatt (1941–2011) was a highly established, peer-nominated expert therapist and was well-regarded by his peers in the disciplines of counseling, psychology, and addiction treatment. Marlatt earned his doctorate in clinical psychology and specialized in the treatment of addictions. He authored or edited 23 books, and published over 300 articles and book chapters (White et al., 2011). He received funding for his funding from both the NIAAA and NIDA, and was honored with numerous prestigious awards including the Innovators award from the Robert Wood Johnson Foundation, the Distinguished Scientific Contribution Award from the Division of Clinical Psychology from the American Psychological Association, and the Lifetime Achievement award from the Association for Behavioral and Cognitive Therapies. Using Hill et al.'s (2017) criteria for evaluating expertise, the researchers were comfortable accepting Marlatt as an identified expert therapist whose skills merited closer examination.

#### Client

At the time of his sessions with Marlatt, Kevin is a male in his 30s who is striving to overcome a crack/cocaine addiction. During the six sessions, Dr. Marlatt helped him to identify high-risk situations and possible triggers for relapse. He also worked with him on the skills for enduring the urges that come with recovery using mindfulness-based relapse prevention. In fact, Kevin had three relapses, which was then processed as part of the therapy. Together they worked to address the shame and guilt that he felt after lapses in abstinence (Marlatt, 2007).

### Measures

#### The specific affect coding system

The Specific Affect Coding System, or SPAFF, was originally developed for the research on the emotional dynamics of marital relationships.^1^ According to Coan and Gottman (2007), SPAFF consists of 20 individual affective behavior codes, including one affect code for neutral behavior, seven positive affect codes (affection, high validation, humor, interest, surprise/joy, low validation and tense humor), and 12 negative affect codes (contempt, belligerence, criticism, stonewalling, defensiveness, high domineering, low domineering, anger, sadness, whining, disgust, and tension; Gottman et al., 1998). Coding is done by trained coders in real-time while watching video recordings of a session, creating a second-by-second data stream of the interaction (Diaz et al., 2023). Following Gottman's research protocol, using Noldus Observer v. 11, each second of the session was assigned a code and each code was weighted; then every 6 s of material was summed to create 150 data points from 900 s (15 min) of video (Gottman et al., 2002). Although SPAFF was initially used for research with marital conflict interactions, it has also been applied to other types of relationships including: parent-baby interactions (Coan and Gottman, 2007; Gottman et al., 2002), the relationship between medical doctors and their patients (Van Walsum, 2005), and most recently, to the therapeutic relationship (Baker et al., 2022; Diaz et al., 2023; Erzar et al., 2012; Luedke et al., 2017; Peluso et al., 2018).

### Procedure

The data used for the current study were the six psychotherapy sessions shown in the films *Relapse Prevention Over Time* (Marlatt, 2007) which were previously recorded. The present study utilized the same procedures for preparing the video data as well as the process for coding the videos using SPAFF coding that were previously reported by Baker et al. (2022) and Diaz et al. (2023). These specific procedures will not be repeated here, though the reader is invited to consult with the previous articles.

#### Dynamical systems mathematical modeling

The DS equations used in this analysis was modified from the work of Gottman and his colleagues (Cook et al., 1995; Gottman et al., 2002) by Peluso et al. (2012).^2^ According to Baker et al. (2022), these differential equations measure changes to a variable of interest (*dT, dC*), over a period of time (*dt*). Mathematically, this relationship is expressed in the form of equations (see Equation 1, below).


(1) $$\begin{array}{l} \frac{dT}{dt}=\;m_{1}T+b_{1}+c_{1}F_{c}\left(C\right)\\ \frac{dC}{dt}=\;m_{2}C+b_{2}+c_{2}F_{T}\left(T\right) \end{array}$$

Each variable in the equation represents either an observed score at a moment in time (in the case of the current project, this would be a score based on SPAFF coding), or a parameter that is a mathematical representation of an element of dynamical system being studied (here, specific dynamics of the therapeutic relationship). According to Baker et al. (2022):

These four parameters (called the *uninfluenced* parameters^3^) are derived using a least squares method, and computed by summing the scores of one partner when the other person is neutral, and compared the changes in scores for each of these at moment *t*+*1*. The *initial state* parameter is derived by total positive and negative scores, when the other person's score is zero (or is having no influence). Broadly speaking, this can be thought of as the individual's unique disposition (positive, negative or neutral), that introduces a constant via the *b*_1_ and *b*_2_ parameters. The *inertia* parameter is “the tendency of remaining in the same state for a period of time” (Cook et al., 1995, p. 114), and is estimated by taking an average of positive scores minus negative scores when the other partner's score was zero.^4^ The greater a person's inertia is, the less likely they are to be open to influence from the other person (pp. 225–226).

In Equation 1, *m*_1_and *m*_2_ are each person's inertia (or their tendency to stay in a previous emotional state), and *b*_1_ and *b*_2_ are the initial state for the therapist and the client. The next parameters, *c*_1_*F*_*T*_*(T), c*_2_*F*_*C*_*(C)* are the influence functions of the therapist on the client, and of the client on the therapist, respectively (Baker et al., 2022; Diaz et al., 2023).

We generated the initial state, inertia, uninfluenced steady state, the thresholds for the influence functions in the negative and positive regimes, as well as the strength and threshold for the repair parameter using Gottman et al.'s (2002) procedure for deriving parameters used in the DS equations.^5^ This provided the necessary parameters to create unique mathematical models for each of the six therapy sessions for both therapists and clients, in accordance with Peluso et al.'s (2012) approach. The key difference in this analysis is that each of the parameters were derived from the weighted and summed SPAFF data coded in each of the six sessions.

#### Data analysis

Similar to Baker et al. (2022) and Diaz et al. (2023), a Kolmogorov-Smirnov Exact test will be used on the percent of time spent in each SPAFF code for both the therapist and the client to explore research questions 1 and 2. This is a non-parametric test that is appropriate for several reasons. First, there were only six observations per variable, and second, the scores themselves were numerically < 5, which made a chi-square (the usual method for investigating) invalid. Next, we chose to compare scores to each other, rather than impose a normal distribution to the scores, as we wanted to see if they differed significantly from one another, from session to session. In instances like the present study, the Kolmogorov-Smirnov Exact test is recommended (Mehta and Patel, 2012). In order to explore research question 3, the DS models will be graphically depicted using phase portraits which will allow for a visual inspection of the relationship dynamics as modeled (Baker et al., 2022; Diaz et al., 2023).

## Results

We begin with an analysis of the individual SPAFF codes for both Alan Marlatt and Kevin that were detected across all six sessions. Next, we present an analysis of the mathematical models of all six sessions, beginning with the model parameters that were derived from the SPAFF data, and then we evaluate the overall dynamics of the relationship at each session using phase-portraits from each of the sessions. Where applicable, all alpha levels were set at 0.05.

### Comparison of affect codes

One of the overarching questions posed in this paper is whether there were any systemic differences in the therapeutic relationship between Alan Marlatt and Kevin as indicated by SPAFF coding of the affect across the sessions. Table 1 lists the number of seconds and the percentage of Marlatt's individual SPAFF codes, as well as the total positive and total negative codes. The number of seconds and percentages of SPAFF codes were compared across the six sessions using a Kolmogorov-Smirnov Exact test.

**Table 1:** SPAFF Codes for Dr. G. Alan Marlatt over six sessions.

**Code**	**Session 1**	**Session 2**	**Session 3**	**Session 4**	**Session 5**	**Session 6**
Low domineering	0 (0)	0 (0)	5 (0.19)	0 (0)	0 (0)	11 (0.41)
Tension	18 (0.67)	24 (0.92)	170 (6.3)	23 (0.85)	96 (3.56)	229 (8.48)
Tense humor	10 (0.37)	0 (0)	47 (1.74)	16 (0.59)	5 (0.19)	45 (1.67)
Sadness	0 (0)	0 (0)	0 (0)	1 (0.04)	0 (0)	0 (0)
Neutral	2456 (90.96)	2310 (88.1)	2024 (74.96)	2466 (91.33)	2317 (85.81)	1935 (71.67)
Interest	36 (1.33)	64 (2.44)	59 (2.19)	30 (1.11)	16 (0.59)	50 (1.85)
Low validation	158 (5.85)	151 (9.33)	260 (4.37)	129 (8.90)	216 (8.89)	212 (7.85)
High validation	13 (0.48)	56 (2.14)	61 (2.26)	30 (1.11)	47 (1.74)	106 (3.93)
Affection	9 (0.33)	3 (0.11)	63 (2.33)	5 (0.19)	0 (0)	103 (3.81)
Humor	0	13 (0.50)	10 (0.37)	0 (0)	3 (0.11)	9 (0.33)
Surprise/joy	0 (0)	0 (0)	1 (0.04)	0 (0)	0 (0)	0 (0)
Total positive	216 (8.0)	287 (11.0)	454 (16.8)	194 (7.1)	282 (10.4)	480 (17.8)
Total negative	28 (0.99)	24 (0.9)	222 (8.2)	40 (1.4)	101 (3.7)	285 (10.5)

Number of seconds per code (Percentage in parentheses).

Looking at Table 1, the SPAFF codes that were detected for Marlatt included: Low Domineering, Tension, Tense Humor, Sadness, Neutral, Interest, Low Validation, High Validation, Affection, Humor, and Surprise/Joy. In addition, we computed the Total Positive and Total Negative scores. The only SPAFF codes that were significantly different from session to session was Low Domineering [*D*_(5)_ = 0.388, *p* < 0.05], Sadness [*D*_(5)_ = 0.492, *p* < 0.05], Affection [*D*_(5)_ = 0.360, *p* < 0.05], and Surprise/Joy [*D*_(5)_ = 0.492, *p* < 0.05]. If we look at Table 1, for the negative codes, Marlatt showed Low Domineering only in sessions 3 and 6, only briefly showed sadness in session 4. For the positive codes, Marlatt showed more Affection in sessions 3 and 6 (2.33% and 3.81%, respectively) than in any other session (all below 1%). As for Surprise/Joy, Marlatt only showed this in session 3, though it was only for 1 s. What may be more interesting is the fact that none of the other SPAFF codes differed significantly from session-to-session, including the Total Positive and Total Negative scores, suggesting a consistency in his emotional expressions with Kevin.

An analysis of the SPAFF codes for Kevin over the span of the six sessions was also conducted. Table 2 lists Kevin's individual SPAFF codes. In addition, just as with Marlatt, we computed both the number of seconds, and the percentages as well as the Total Positive and Total Negative scores. Unlike Marlatt's SPAFF codes, Kevin's scores did show significant differences from session to session for multiple SPAFF codes including: Disgust [*D*_(5)_ = 0.500, *p* < 0.05], Contempt [*D*_(5)_ = 0.667, *p* < 0.05], Low Domineering [*D*_(5)_ = 0.500, *p* < 0.05], Tension [*D*_(5)_ = 0.430, *p* < 0.05], Neutral [*D*_(5)_ = 0.543, *p* < 0.05], Interest [*D*_(5)_ = 0.500, *p* < 0.05], High Validation [*D*_(5)_ = 0.424, *p* < 0.05], Affection [*D*_(5)_ = 0.500, *p* < 0.05], and Surprise/Joy [*D*_(5)_ = 0.667, *p* < 0.05]. Neither Total Positive or Total Negative were significantly different, however. Looking at the negative SPAFF codes in Table 2, Disgust and Low Domineering was present in sessions 3, 4, and 6, while Contempt was in sessions 4 and 6. Tension was present in every session, but what was notable was the range from a low of 5.3% in session 4 to 19.5% in session 3. Concomitant with this finding, Neutral ranged from 86.8% in session 4 to 57.1% in session 3. Looking at the positive codes, Kevin showed Interest in sessions 1, 3, and 6, High Validation in every session except session 2, Affection in sessions 3, 5, and 6 only, and Surprise/Joy in sessions 3 and 6. Considering that Kevin had relapses between sessions 1 and 2, between sessions 3 and 4, and between sessions 5 and 6 it is noteworthy the changes across the six sessions, which will be discussed further below.

**Table 2:** SPAFF codes for Kevin over six sessions.

**Code**	**Session 1**	**Session 2**	**Session 3**	**Session 4**	**Session 5**	**Session 6**
Disgust	0	0	1 (0.01)	1 (0.01)	0	1 (0.01)
Contempt	0	0	0	1 (0.01)	0	9 (0.03)
Low domineering	0	0	39 (1.44)	13 (0.72)	0	24 (1.0)
Criticism	0	0	4 (0.10)	0	0	0
Anger	0	207 (7.9)	87 (3.2)	21 (1.2)	178 (6.6)	78 (2.7)
Tension	247 (9.14)	178 (6.8)	529 (19.5)	95 (5.3)	165 (6.1)	252 (9.3)
Tense humor	9 (0.33)	9 (0.34)	42 (1.5)	9 (0.5)	9 (0.3)	51 (1.9)
Defensive	0	0	9(0.03)	3 (0.01)	5 (0.01)	30 (1.1)
Sadness	56 (2.1)	212 (8.0)	373 (13.8)	71 (4.0)	457 (17.0)	374 (14.0)
Neutral	2,232 (82.7)	1,997 (76.1)	1,543 (57.1)	1,564 (86.8)	1,848 (68.4)	1,809 (67.0)
Interest	33 (1.2)	0	3 (0.1)	0	0	2 (0.1)
Low validation	111 (4.1)	15 (0.1)	2 (0.1)	0	13 (0.5)	13 (0.5)
High validation	12 (1.2)	0	18 (0.7)	9 (0.5)	15 (0.5)	22 (0.8)
Affection	0	0	31 (1.1)	0	6 (0.2)	26 (0.9)
Humor	0	3 (0.1)	15 (0.5)	13 (0.7)	4 (0.1)	11 (0.4)
Surprise/joy	0	0	4 (0.1)	0	0	3 (0.1)
Total positive	156 (5.8)	18 (0.6)	73 (2.7)	22 (1.2)	38 (1.4)	77 (2.9)
Total negative	312 (11.5)	606 (23.1)	1,084 (40.1)	214 (11.9)	814 (30.1)	814 (30.1)

Number of seconds per code (percentage in parentheses).

#### Comparisons of “meta” states

In order to organize the individual SPAFF codes into functional groupings beyond total positive, total negative and neutral, we have divided the positive and SPAFF codes into two sub-groups. Looking at the positive SPAFF codes, there are codes that are expressions of positive affect, and those that are relationship focused. The positive affect codes include Affection, Humor and Surprise/Joy. The positive relationship-focused codes seem to be facilitative of the relationship and include Low Validation, High Validation, and Interest. In terms of the negative SPAFF codes, again there are codes that are expressions of negative affect, and those that are relationship focused. The negative affect codes include Anger, Sadness, Whining, Disgust, Contempt, Tension, and Tense Humor. The negative relationship-focused codes tend to be more controlling of the relationship and include Belligerence, Low Domineering, High Domineering, Criticism, Defensive, and Stonewalling. Thus, we combined the percent of time for each of the constituent codes and created four new “meta” states of Positive Affect, Facilitating, Negative Affect and Controlling for both Marlatt and Kevin. Across the six sessions, Marlatt's Control [*D*_(5)_ = 0.667, *p* < 0.05], and Positive Affect [*D*_(5)_ = 0.486, *p* < 0.05] states significantly changed over the six sessions. Looking at Table 3 Marlatt shows Control in only the 3rd and 6th sessions, and only for 5 and 11 s, respectively. He shows Positive Affect in all six sessions, but in session 3 he shows it nearly 3% of the time, while in all of the others, it is < 1 percent. For Kevin, only his Facilitative state significantly changed over the six sessions [*D*_(5)_ = 0.395, *p* < 0.05]. Looking at Table 4, Kevin shows Facilitative codes in all six sessions, but shows the most (5.8% of the time) in session 1. It is possible that given the nature of substance abuse treatment he was engaging in impression management more in the first session, and that subsequently (as relapses occurred), he engaged in less of this behavior.

**Table 3:** Meta-States SPAFF codes for Dr. G. Alan Marlatt over six sessions.

**Code**	**Session 1**	**Session 2**	**Session 3**	**Session 4**	**Session 5**	**Session 6**
Negative affect	28 (1.0)	24 (1.0)	217 (8.0)	40 (1.4)	101 (3.7)	274 (10.1)
Control	0 (0)	0 (0)	5 (0.1)	0 (0)	0 (0)	11 (0.3)
Neutral	2,456 (90.96)	2,310 (88.13)	2,024 (74.96)	2,466 (91.33)	2,317 (85.81)	1,935 (71.67)
Facilitate	207 (7.7)	271 (10.3)	380 (14.1)	189 (7.0)	279 (10.3)	368 (13.6)
Positive affect	9 (0.3)	16 (0.6)	74 (2.7)	5 (0.2)	3 (0.1)	112 (0.4)

Number of seconds per code (percentage in parentheses).

**Table 4:** Meta-states SPAFF codes for Kevin over six sessions.

**Code**	**Session 1**	**Session 2**	**Session 3**	**Session 4**	**Session 5**	**Session 6**
Negative affect	312 (11.5)	606 (23.1)	1,032 (38.2)	198 (11.0)	809 (30.0)	760 (28.1)
Control	0	0	52 (2.0)	16 (1.1)	5 (0.1)	54 (2.0)
Neutral	2,232 (82.7)	1,997 (76.1)	1,543 (57.1)	1,564 (86.8)	1,848 (68.4)	1,809 (67.0)
Facilitate	156 (5.8)	15 (0.6)	23 (0.8)	9 (0.5)	28 (1.0)	37 (1.3)
Positive affect	0	3 (0.1)	50 (1.9)	16 (0.7)	10 (1.0)	40 (1.3)

Number of seconds per code (percentage in parentheses).

In considering these results viz. Research Question 1, in which we sought to investigate how the emotional expression of the therapist and the client change across the entire course of therapy for substance abuse, in terms of coded emotional expression in sessions over time for this particular series of therapy sessions, the therapist displayed fewer emotion codes than the client, and that relative to Kevin, only a few of Marlatt's codes vary significantly over the course of the six sessions. At the same time, Kevin did show significant changes in a number of codes over the six sessions. However, to assess the dynamic nature of the relationship, and the impact of this on the overall system, we will consider the results of the mathematical modeling next.

### Mathematical modeling of the therapeutic relationships

#### Comparisons of parameters across sessions

Following the procedure laid out by Baker et al. (2022) and Diaz et al. (2023) using Peluso et al.'s (2012) equations, mathematical models and parameters for all six sessions were computed. Table 5 lists the derived parameters for all six sessions for Alan Marlatt and Table 6 lists the parameters for Kevin. Again, just as before, to determine if the parameters differed significantly from session-to-session a Kolmogorov-Smirnov Exact test was employed. For both Alan Marlatt and Kevin, the parameters of their models were not significantly different from one another, suggesting that despite the differences detected in Kevin's individual SPAFF codes described above, the model parameters for both Marlatt and Kevin are relatively consistent across the sessions over time. This is consistent with the findings of Diaz et al. (2023) when they compared six video recorded sessions with another expert therapist and client. At the time, they suggested that “the parameters are derived primarily from summed scores when one partner or another is negative or positive” (p. 8). As with their previous findings, neither Marlatt or Kevin's total positive or total negative scores were significantly different. However, unlike findings by Baker et al. (2022) and Diaz et al. (2023), Table 6 reveals that for Kevin, several sessions did not have a negative threshold parameter estimated from the SPAFF data. As a result, the influence function of the therapist on the client is a bi-linear, rather than a tri-linear function. Hence, the phase portraits will have fewer attractor points, or options for the session to eventually wind up. So, considering Research Question 2, which sought to understand how the dynamics of the relationship for both the therapist and client change across the entire course of substance abuse therapy, while the model parameters were not significantly different from session to session, there may be dynamic variations between the sessions that are worth exploring via a phase-space portrait.

**Table 5:** Dynamical systems model parameters for G. Alan Marlatt.

	**a2**	**r2**	**UnSS**	**nth**	**pth**	**kr**	**sr**
Session 1	−0.1324441	0.3611994	−0.2073325	−1.6	0.6	−1	1.2
Session 2	0.1408405	0.307107	0.20326443	−3.8	−1.3	−2.5	6.1
Session 3	−0.2510611	0.2716847	−0.3447149	−5	−3	N/A	N/A
Session 4	−0.1201938	0.07043698	−0.1293014	−1.1	−0.5	N/A	N/A
Session 5	−0.5614022	0.2544554	−0.7530095	−2.7	0	−2.6	3.3
Session 6	0.1218353	0.6107724	0.31301814	−6	−1.8	−2.7	3

a1, Initial State; r1, Inertia; UnSS, Uninfluenced Steady State; nth, Influence Function Negative Threshold; pth, Influence Function Positive Threshold; kr, Threshold of Repair; sr, Strength of Repair; N/A, parameter could not be estimated.

**Table 6:** Dynamical systems model parameters for Kevin.

	**a2**	**r2**	**UnSS**	**nth**	**pth**	**kr**	**sr**
Session 1	0.1652314	0.4301383	0.28995	N/A	0.2	−2.7	1.9
Session 2	−0.7105349	0.714605	−2.4896543	N/A	−0.2	−9.5	0.5
Session 3	−0.6360675	0.72216	−2.2893302	−2.6	−1	N/A	N/A
Session 4	−0.4974667	0.4330673	−0.8774705	N/A	−1	N/A	N/A
Session 5	−0.559131	0.7634271	−2.3634617	−1.2	0.2	N/A	N/A
Session 6	−1.038966	0.4618898	−1.9307681	−0.6	0.6	N/A	N/A

a2, Initial State; r2, Inertia; UnSS, Uninfluenced Steady State; nth, Influence Function Negative Threshold; pth, Influence Function Positive Threshold; kr, Threshold of Repair; sr, Strength of Repair; N/A, parameter could not be estimated.

#### Phase portrait visualization of the therapeutic relationships

**Figure 1:**
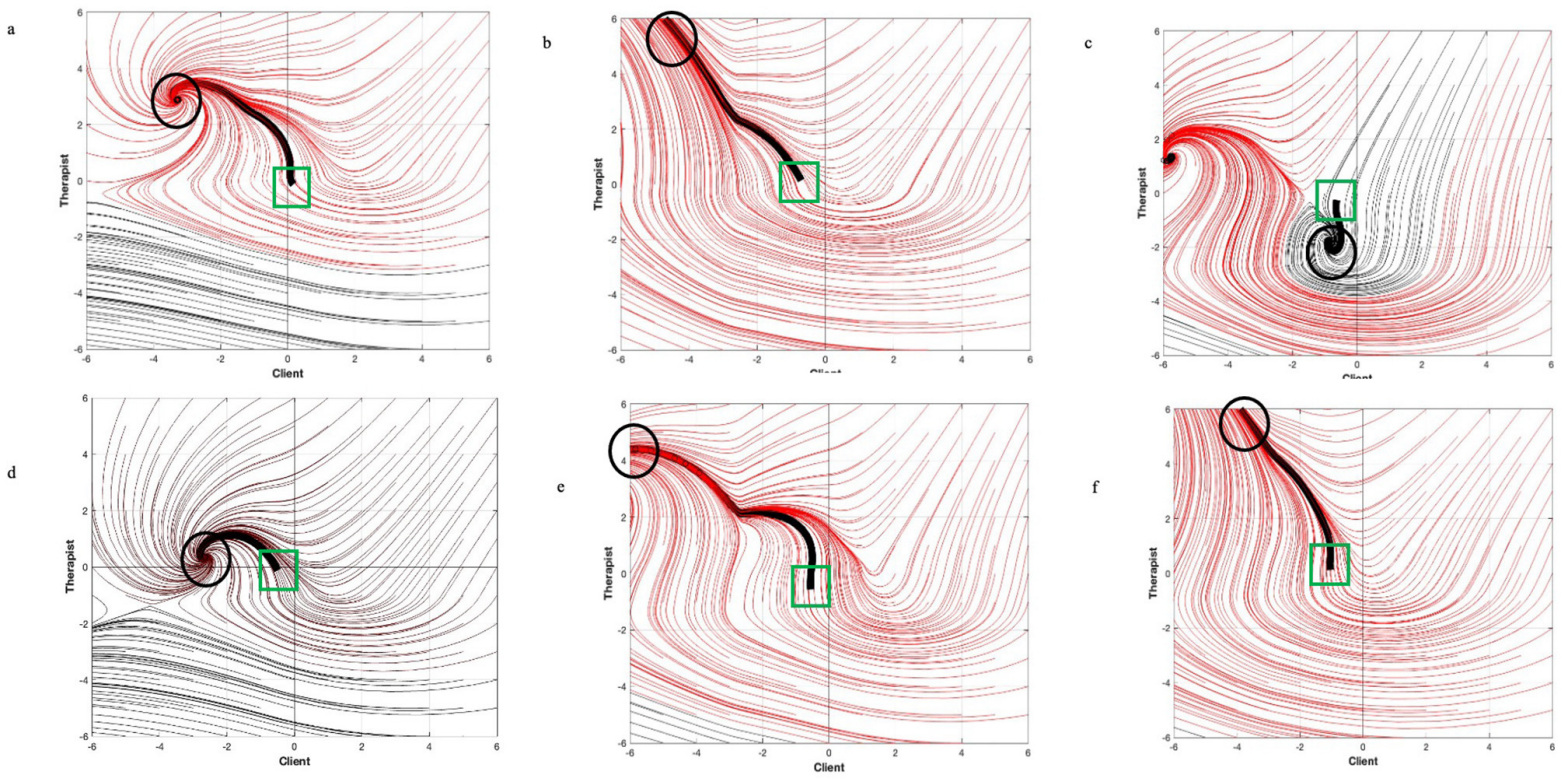
Phase portraits of all six sessions of therapy between Alan Marlatt and Kevin Client. **(a)** Session 1. **(b)** Session 2. **(c)** Session 3. **(d)** Session 4. **(e)** Session 5. **(f)** Session 6. Beginning coordinates denoted by green squares, and ending coordinates denoted by black circles. Black line is the trajectory that the session likely took based on the starting coordinates.

The models in the current study were derived from the SPAFF data of the emotional expression in the six sessions of real therapy with Alan Marlatt and his client Kevin taken from the *Psychotherapy Over Time* video series (Marlatt, 2007). The parameters that are derived from the mathematical models are best considered using a graphic visualization, especially for complex systems (Liebovitch et al., 2011). The phase-space portraits in Figure 1 shows two-dimensional phase-space portraits of all six sessions and were created using the parameters derived from the differential equations (see Equation 1, above listed in Tables 5, 6). This was the result of an iterative process using 10 time-steps and displays the trajectory lines for every set of all potential starting coordinates for the system, as well as the critical point(s) in the system (see Baker et al., 2022; Diaz et al., 2023 for more information). Once the system is created, then using the initial starting point for the session, the projected “trajectory” of the session can be estimated. This is accomplished in each of the six sessions in Figure 1 by averaging the first 10 percent of the SPAFF codes for therapist and client. In each of the six phase portraits in Figure 1 these coordinates (client starting point value on the *x*-axis, therapist starting point value on the *y*-axis) are indicated by a green square. The estimated trajectory of the actual sessions are indicated on the phase-space portrait (as a black line) to illustrate how the math model predicted the quality and endpoint of the relationship in the session where the parameters for the whole system were derived. The quadrant in which the critical points are located is an indication of the quality of the relationship (e.g., a positive-positive quadrant vs. negative-negative quadrant), while the black trajectory line represents the estimated actual endpoint of the therapeutic relationship (Baker et al., 2022; Diaz et al., 2023; Gottman et al., 2002; Liebovitch et al., 2011; Peluso et al., 2012). The endpoint coordinates of the trajectory in each of the phase portraits in Figure 1 is denoted by a black circle.

There are several aspects of the phase-space portraits for the six sessions as in Figure 1 to note. First, in all the sessions there are attractors in the therapist positive-client negative quadrant. This has been identified as a “working” quadrant where the client is struggling and the therapist is providing support (Baker et al., 2022; Diaz et al., 2023; Peluso et al., 2012), though it is not as optimal as an attractor in the positive-positive quadrant. Indeed, in the six sessions, Kevin had three relapses and struggled to deal with these, which is reflected in these models (this will be discussed in greater detail, below). However, each of these models are created uniquely by the parameters generated from the SPAFF scores for that given session. In these cases, an attractor in that quadrant represents the “best case scenario” for that session. So, while we can estimate the trajectory of that session based on the starting point, in five of the six sessions, the final attractor was in the therapist positive-client negative quadrant. Moreover, with the exception of the third session, the projected trajectory led to the attractor in that quadrant. In the third session (which was notable for Kevin's high level of Tension and low level of Neutral, see above). In the third session, it is notable that there was another attractor in the therapist negative-client negative quadrant (there are attractors in that quadrant that are toward the lower left corner, which is usually indicative of a poor session outcome). In the session with Kevin, the additional attractor could be reflective of the increased Tension and decreased Neutral in that session.

In terms of Research Question 3, which was an investigation of how the overall therapeutic relationship change across the entire course of therapy, while the majority of the parameters of the models did not significantly differ from one another, an examination of the phase-space portraits shows vastly different dynamics at work in each of the sessions. At the same time, the overwhelming prevalence of attractors in the therapist positive-client negative quadrant leads to several conclusions: (1) these were the best options for the session, and often the session was attracted to that outcome, (2) as the similarities of the sessions to each other, is expected given the lack of significant difference, and (3) when compared to the findings of Baker et al. (2022) and Diaz et al. (2023), which looked at generic psychotherapy, the models for Marlatt's treatment of Kevin's substance use disorder attests to the persistent emotional “work” that is done, even by expert therapists. The implications for this will be discussed below.

## Discussion

The present study integrated qualitative Specific Affect Coding System SPAFF coding and quantitative dynamical systems (DS) modeling to analyze observational measures of emotional expression between the therapist (Dr. G. Alan Marlatt) and the client (Kevin) through a course of six psychotherapy sessions with a primary therapeutic focus on relapse prevention using mindfulness-based relapse prevention treatment. The analysis of SPAFF coding revealed notable differences in emotional expression between Marlatt and Kevin across the sessions. Marlatt displayed consistent emotional expressions with minimal variation, while Kevin exhibited significant fluctuations, presumably in response to his struggles with addiction and episodes of relapse. However, the study found no significant differences in model parameters between sessions, indicating consistency despite changes in individual SPAFF codes. These findings underscore the complex interplay of emotions within the therapeutic relationship, reflecting the challenges inherent in addressing substance use disorders and relapse prevention. Moreover, the mathematical modeling of the therapeutic relationship provided insights into the underlying dynamics between Marlatt and Kevin.

Although the total positive and negative scores suggest consistency in emotional expression toward Kevin, several SPAFF codes differed from session to session. Dr. Marlatt showed both Low Domineering and Affection in sessions 3 and 6; he showed Sadness in session 4 and briefly Surprise/Joy in session 3. Meta-states revealed that Marlatt exhibited control in sessions 3 and 6, and showed positive affect in all sessions, with the highest percentage in session 3. The analysis of Kevin's SPAFF codes revealed significant differences from session to session. Low Domineering and Disgust were present in sessions 3, 4, and 6. Contempt was present in sessions 4 and 6. Tension was detected in all sessions and had the highest percentage of occurrence in session 3. Kevin showed high validation in sessions 1, 3, 4, 5, and 6. He showed interest in sessions 1, 3, and 6. Affection was present in sessions 3, 5, and 6 and Surprise/Joy in sessions 3 and 6. However, neither the total positive and negative codes were significantly different across sessions.

The content of the sessions can explain the differences in SPAFF codes between Marlatt and Kevin. Kevin relapsed several times throughout the treatment process, most notably between sessions 1 and 2, between sessions 3 and 4, and between sessions 5 and 6. Sessions 1 and 2 occurred 4 months apart rather than 1 month apart as initially planned. Among the people who met criteria for a substance use disorder, ages 18 and older, approximately 85% did not attend treatment with many endorsing not feeling ready to attend treatment and fearing judgment from others (Substance Abuse Mental Health Services Administration, 2019) demonstrating the need for treatment personnel to create a safe environment where clients can feel encouraged to address their presenting problems. A question arises concerning what enabled Kevin to share with Marlatt about his relapse so early in the therapeutic relationship, rather than avoiding the session entirely. During session 1, Marlatt utilized interest to prompt Kevin to share about his history. Through this process, Marlatt was able to elicit Kevin's upbringing including his mom's previous struggles with cocaine use and his father failing to be present during his childhood. During the same session, Kevin shared that he had a lapse 3 weeks prior to therapy beginning (SPAFF code in *italics*):

Marlatt: So you were off until then you had just a one-day lapse, we might call it? –(*Interest*)
Kevin: “Yeah.”
Marlatt: “What happened then (30:35, 2007)?” – (*Interest*)
Kevin: I don't know. I went home and found a check in the mail that had come to me (30:26- 30:42, 2007).

Marlatt acknowledged that this was a lapse, rather than an ongoing pervasive relapse, demonstrating relapse prevention and also inviting him to explore the event in a non-judgmental way. Of note, in session 1, was the significant difference of Kevin's use of the “meta” facilitative code, which may have served as a protective strategy for Kevin against facing potential discrimination as a black, male and as someone who struggled against substance use. Marlatt's use of interest, and lack of facilitative codes within this session, may have increased Kevin's comfort and ability to trust Marlatt.

In session 3, Kevin was reflecting on moving into a recovery house where another tenant had relapsed, having to raise his younger brother (as a child) due to his mom's drug use, and his conflicting feelings surrounding turning 36 years old. From a mindfulness-based relapse-prevention approach, Marlatt did not stigmatize or devalue Kevin for sharing about his progression of substance use. Regarding Marlatt's increased use of low domineering and the “meta” state of control, in session 3, Marlatt may have therapeutically needed to utilize redirection as a means of shifting Kevin's perceptions surrounding his negative views of himself. To offset this, Marlatt may have used additional positive affect, which increased to 3% in session 3 in an attempt to ensure he is continuing to validate Kevin's emotional difficulties. Graphically, in Figure 1c, the attractor point was in the negative-negative quadrant, because Marlatt's and Kevin's initial state parameters were negative (−0.25 and −0.063, respectively). While this is not the best quadrant for a session's dynamic, Marlatt's use of affect may have been a result of attempting to help Kevin in managing what he described as a pre-lapse in the session (again, SPAFF code in *italics*).

Kevin: I guess I'm in pole position or something and I'm waiting for -for something to happen the only way for it to actually just to see it actually happen is to stay waiting in pole position (…) (26:4, 2007).
Marlatt: (…) sounds pretty impressive just thinking about it that way (*Affection*) because you're part of it is just me you you've said how self-critical you are and things that have happened in the past and this being able to sit there and experience what things are like at the pole can be moving toward more acceptance (*High Validation*) (27:19, 2007)
Kevin: Yeah
Marlatt: and uh that that is also happening
Kevin: Yeah
Marlatt: because you're not putting yourself back in the same place that you were before when you were feeling that way (*Affection*) (27:30, 2007).

This is a good example of the SPAFF code of affection, where Marlatt utilized an opportunity to compliment Kevin's actions and progress thus far in his recovery. Affection is also one of the codes embedded in the Positive Emotion meta state described above.

Session 4 occurred 2 months after session 3 potentially due to Kevin's relapse following session 3. The “tension reduction hypothesis” posits that those with substance use disorders utilize substances to temporarily reduce tension and provide them with relief reinforcing a self-defeating cycle of addictive behaviors (Marlatt, 2006). Interestingly, tension was highest for Kevin in session 3, just before he relapsed, and then his levels of tension were lowest in session 4. Marlatt's inertia parameter (i.e., his likelihood to move from one emotion state to another) was the lowest of the six sessions (0.07), and Kevin's was moderate (0.46), suggesting that Marlatt might have been moving in and out of emotional states, and that (relative to his other sessions), Kevin was equally receptive to it. Recognizing increases in tension, such as psychomotor agitation and/or speech disturbances, may provide helpful insight for therapists to predict future relapses based on affect. Marlatt theorized that mindfulness based interventions could help provide another source of relief to individuals rather than them having to resort to substance use (Witkiewitz et al., 2005). In session 6, Marlatt demonstrated increased affection and low domineering in addition to significant differences in the “meta” state of control codes (which encompasses low domineering). In addition, Marlatt's initial state parameter was positive (0.12), and his inertia parameter was at its highest (0.61). This lends further credence to the observation that he was staying with a more positive affect. Kevin demonstrated significant changes in the use of contempt, disgust, and low-domineering, potentially due to relapsing again between sessions 5 and 6. In addition, his initial state parameter for the session was at its *lowest* (−1.0), so Marlatt's use of low-domineering may have been an attempt to either influence him away from this negative affect, or to “wrap-up” the therapeutic process. Marlatt acknowledged, through relapse prevention, the cyclical nature of addiction includes clients often becoming shameful surrounding their relapse which reinforces addictive behavior (Witkiewitz and Marlatt, 2004). Marlatt's increased use of affection could have reduced shame for Kevin through promoting a sense of we-ness (us-against-the-addiction rather than me-against-you). This may have ultimately enabled Kevin to end on a positive note, through his use of affection and surprise/joy, which demonstrated significant differences.

Through individual SPAFF codes, relapse prevention, and harm reduction, Marlatt can be seen prioritizing a therapeutic relationship with Kevin from the initial psychotherapy session. Marlatt's approach to therapy further reinforced the common factor theory of the therapeutic relationship (Baker et al., 2022; Norcross and Lambert, 2019; Wampold and Imel, 2015). As a expert-therapist, Marlatt seamlessly integrated the principles of mindfulness-based relapse prevention but his use of emotional expression and consistency is the common factor that appeared to assist Kevin in continuing therapy. In one of the post-session interviews, Marlatt acknowledges that despite Kevin's strengths, Kevin still experienced self-doubt, which could contribute to subsequent lapses and could have contributed to changes among his initial state parameters and inertia from session to session. Marlatt's use of low-domineering, while considered a “negative code,” appeared to be in an attempt to help Kevin keep the focus of the session and/or shift self-defeating patterns rather than to patronize or lecture. Kevin's struggles with addiction, as evidenced by SPAFF codes and trajectory patterns, signify the importance of adaptive interventions and ongoing support in the treatment process. Additionally, the results of this study suggest that the relational dynamic evolved as therapeutic alliances improved, which was suggested by the presence of affection and high validation in later sessions.

Dr. Marlatt shows consistency in his emotional expressions with Kevin throughout all six sessions, providing a sense of stability to Kevin. Marlatt actively assisted Kevin in identifying triggers, developing coping strategies, and addressing feelings of guilt and shame often associated with relapses. Graphically, in Figure 1, this is highlighted by the fact that five of the six sessions had attractor points in the therapist positive, client negative quadrant. He processed Kevin's relapses as part of the therapy. In particular, Dr. Marlatt collaborated with Kevin in identifying high-risk situations and possible triggers for relapse, as well as in developing appropriate strategies to combat his enduring urges common in the recovery process.

### Clinical takeaways and implications

Substance use disorders involve psychological, emotional, and behavioral components, requiring a comprehensive treatment approach. The relational dynamic observed in the six therapy sessions further emphasizes the complexity of treating substance use disorder (SUD). Understanding the complex interaction of emotions and relational dynamics within a therapy setting can improve the development of tailored interventions and enhance therapeutic outcomes. Within substance use treatment, it is imperative that therapists remain flexible due to the unpredictable nature of substance use disorder. For example, due to Kevin's lapses, the spacing between sessions did not follow the monthly sessions that Marlatt and Kevin initially agreed upon. Rather, session 2 occurred 4 months after session 1, session 4 occurred 2 months after session 3, and session 5 occurred the same day as session 4. Marlatt demonstrated the importance of continuing to work with Kevin, despite treatment not following a linear process, which ultimately enabled Kevin to continue receiving care and process his lapses. Lapses are relatively common in addiction treatment, and therapists must remain supportive of clients throughout these challenges; “confrontational” methods associated with substance use treatment (Substance Abuse Mental Health Services Administration, 2019) encourages emotional dynamics such as low-domineering, criticism, and contempt. Negative affect codes and negative relationship-focused codes in a therapy session would negatively impact the therapeutic relationship and thus treatment itself, regardless of the approach used. Marlatt used Kevin's lapses as opportunities to build the therapeutic relationship through his affect. For instance, Dr. Marlatt encouraged Kevin by saying, “I just want to re-emphasize the progress that you have made since we were together last time, and you are—your recognition of the risks and things like that; the pre-lapse kinds of things are very important because each time you're getting a sense of what you need to do and how to get whatever help you might need it before that it gets to that point” (Marlatt, 2007, 41:46, session 3). This interaction could have become emotionally volatile, even if the same theoretical orientation was used, through the use of low-domineering such as inadvertently communicating Kevin's lapse is a moral failing (i.e., taking on a lecturing role for why he should or should not think, feel, and behave a certain way).

Marlatt's approach to relapse prevention emphasizes the importance of client-centered care, individual treatment goals, and utilization of cognitive-behavioral and mindfulness-based prevention strategies in addiction treatment in conjunction with consistent emotional expression. Marlatt's response exemplified his commitment to fostering a non-judgmental and supportive therapeutic environment, where setbacks were used as opportunities to utilize interest, affection, and validation and/or keep a neutral stance to enable Kevin to manage his emotional state. By maintaining a curious and empathetic stance, Marlatt reinforced the therapeutic alliance and affirmed Kevin's agency in his recovery journey. It should be noted that while Marlatt did use low-domineering, through interruptions, it appeared to be in an attempt to “steer” Kevin toward positive affect rather than to lecture or patronize Kevin. Therapists should engage in ongoing evaluation and adaptation of treatment approaches based on client needs, progress, and therapeutic outcomes. Flexibility and responsiveness to client needs are crucial in optimizing treatment effectiveness.

### Therapist training specific to SUD

Increased emotional reactivity is specific to those with a substance use disorder, due to the higher percentage of severe and/or any mental illness among the population (Substance Abuse and Mental Health Services Administration, 2023), risk of post-acute withdrawals, and difficulties with emotional regulation. Ultimately, a novice therapist may struggle to manage a client's intense emotional affect in addition to navigating their own biases surrounding substance use due to people in recovery often being viewed as possessing a moral failing (Substance Abuse Mental Health Services Administration, 2019). Being in tune with microexpressions, may allow therapist's-in-training to have more tangible ways of reading and responding to the emotional affect of those with substance use disorders (i.e., noticing eyebrows moving up as an indicator of sadness or one lip corner moving upward to demonstrate contempt). Marlatt's ability to respond to Kevin's shifting emotional variability whether it were expressions of frustration or shame-reflects a therapeutic sensitivity that may be especially important in substance use disorders treatment. McLellan et al. (2000) found that personalized care, which takes into account the client's emotional regulation capabilities, leads to better outcomes in relapse prevention. Emotional attunement from a therapist enables the client to feel validated, ultimately fostering a stronger therapeutic alliance. Moreover, consistent emotional regulation from the therapists, as seen in Marlatt's demeanor, has been shown to create a safe and non-judgmental environment, a core component in addiction recovery (Crits-Christoph et al., 2011).

Marlatt's approach to Kevin, a Black male, also highlights the relevance of culturally competent care in SUD treatment. While Kevin's racial identity was not explicitly addressed in session dialogues, researchers found that clients from marginalized backgrounds often experience higher rates of stigma and discrimination, both within the healthcare system and society (Williams and Mohammed, 2009). This can impact the client's willingness to engage in therapy, making the therapist's emotional attunement and non-judgmental stance even more crucial. Marlatt's calm and consistent demeanor, particularly when addressing Kevin's relapse, reflects an implicit understanding of the need for a safe therapeutic space, which can be especially important for clients from historically marginalized and underserved communities. Incorporating cultural competence in SUD treatment improves engagement and treatment outcomes for clients from diverse backgrounds (Guerrero et al., 2013).

### Limitations and future directions

This study was constrained to six therapy sessions, potentially limiting the generalization of the findings. The small sample size may have also limited the power of statistical analyses. Future studies would benefit from the use of measures such as the working alliance inventory, quality of life questionnaire, and addiction severity index to further clarify the impacts of emotional expression on these factors. Additionally, outcome measures of symptom reduction or clinical success could provide additional validation for the models and their parameters, that were unavailable with the current study. Comparisons of the initial states and inertia parameters, as well as the phase portraits that are created in the early stages of therapy could be compared to the later stages of therapy for cases where there were clinically significant gains and cases where there were not.

Like Diaz et al. (2023) and Baker et al. (2022), the code that had the largest percent of time was Neutral. Neutral happens when a person is listening to another person, or when a person is speaking without any other emotional characteristics. It is also used as a “default” code when there are moments that do not fit any of the codes (Gottman et al., 1998). This study attempted to create “meta” states (Facilitative, Control, Positive and Negative Emotion) to combine individual SPAFF codes and potentially create larger percentages of time. However, Neutral still remained the large component of the affect data. Echoing the suggestions of Diaz et al. and Baker et al. future studies should leverage technology in machine learning, vocalization detection, and affective computing that could shed additional information from these segments of time.

Future research should replicate the study with a larger sample size and investigate long-term therapeutic relationships to gain a deeper understanding of the dynamics of therapeutic alliance. In terms of the mathematical modeling, assessing the validity of the bi-linear or tri-linear influence functions (relative to other representations) would be helpful, given the findings reported herein. Additionally, research examining therapeutic processes and outcomes across diverse client populations, including varying cultural, socioeconomic, and demographic backgrounds, is suggested to improve culturally competent treatment and enhance treatment efficacy for all clients. Moving forward, a deeper understanding of these dynamics can enhance the efficacy of relapse prevention interventions and improve outcomes for individuals undergoing substance use treatment. Future studies may benefit from exploring if inconsistencies within session timings impact the progress within the therapeutic relationship.

## Conclusion

The present study contributes to the growing literature on therapeutic alliance and the dynamics within addiction treatment. By integrating qualitative and quantitative methodologies, the research offers a comprehensive understanding of the dynamic relational changes throughout treatment, revealing the complexities of substance use treatment. In *Relapse Prevention Over Time*, Dr. Marlatt demonstrated his therapeutic approach to helping clients with addictions with prevention or coping with relapses. In this six-session series, Dr. Marlatt helps his client, Kevin, who is struggling with crack-cocaine addiction, how to recognize and handle high-risk situations and potential relapse triggers by teaching Kevin skills to get through these situations. The results of this study emphasize the need for therapists to adapt dynamically to clients' evolving emotional states. Additionally, utilizing dynamic systems modeling offers a novel approach to examining the intricacies of therapeutic relationships, providing valuable insight into the change process. The prevalence of attractors in the therapist positive-client negative quadrants suggests the importance of strong therapeutic alliance and supportive environment in SUD treatment, indicating that clients struggling with addiction issues need consistent support and understanding from their therapists. Furthermore, counselors should prioritize emotional attunement and foster an empathetic, validating environment for clients to share about lapses rather than feeling the need to hide them. The study underscores the importance of the integration of therapeutic modalities in addiction treatment. Throughout the six sessions, Marlatt integrated cognitive-behavioral techniques, mindfulness-based practices, and other modalities, allowing for a tailored intervention that addressed Kevin's complex needs during his addiction treatment.

## Data Availability

The raw data supporting the conclusions of this article will be made available by the authors, without undue reservation.

## Footnotes

^1^For a more thorough explanation of SPAFF coding applied to the therapeutic relationship, please consult Baker et al. (2022) and Diaz et al. (2023).

^2^Please see Peluso et al. (2012) and Baker et al. (2022) for a more detailed discussion of DS modeling applied to the therapeutic relationship.

^3^These two parameters are combined to created uninfluenced steady state is created by combining the inertia parameter (resistance to change), and the initial state (the individual's dispositional characteristics) using the formula: *b*_1_*/m*.

^4^For specific information on how the parameters are derived, please see Gottman et al. (2002).

^5^Several programs are available to accomplish this, the present study used such as the “dyad” package written in the R programming language, and freely available (Madhyastha and Hamaker, 2009).
